# WHO MAY BENEFIT FROM ROBOT-ASSISTED GAIT TRAINING WITH AN EXOSKELETON IN SUBACUTE STROKE PATIENTS? A PRESPECIFIED ANALYSIS

**DOI:** 10.2340/jrm.v58.45822

**Published:** 2026-06-17

**Authors:** Won Hyuk CHANG, Tae-Woo KIM, Hyoung Seop KIM, Fazah Akhtar HANAPIAH, , Jong Weon LEE, Seung-Hyeon HAN, Chai Wen JIA, Dae Hyun KIM, Deog Young KIM

**Affiliations:** 1 Department of Physical and Rehabilitation Medicine, Center for Prevention and Rehabilitation, Heart Vascular and Stroke Institute, Samsung Medical Center, Sungkyunkwan University School of Medicine, Seoul, Republic of Korea; 2 TBI Rehabilitation Center, National Traffic Injury Rehabilitation Hospital, Gyeonggi-do, Republic of Korea; 3 Department of Rehabilitation Medicine, Seoul National University College of Medicine, Seoul, Republic of Korea; 4 Department of Rehabilitation Medicine, Pohang Stoke and Spine Hospital, Pohang, Republic of Korea; 5 Faculty of Medicine, Universiti Teknologi MARA, Selangor, Malaysia; 6 Department and Research Institute of Rehabilitation Medicine, Yonsei University College of Medicine, Seoul, Republic of Korea; 7 Department of Psychology, Faculty of Social Sciences, Raffles University, Johor, Malaysia

**Keywords:** stroke, ambulation, rehabilitation, robot therapy, robot-assisted gait training, overground gait training

## Abstract

**Objective:**

To identify factors associated with the achievement of independent gait after the robot-assisted gait training (RAGT) with an exoskeletal wearable robot in subacute stroke patients.

**Design:**

An international, multicentre, randomized, controlled trial.

**Subjects/Patients:**

This prespecified analysis was performed on 58 and 69 subacute stroke patients in the RAGT and control groups.

**Methods:**

Each RAGT and the conventional gait training was provided 5 times per week for a period of 4 weeks. A Functional Ambulation Categories score of > 3 immediately post-intervention was defined as independent ambulation and clinical significance. Univariate and multivariate binary logistic regression models were used to determine possible predictors of clinically significant response to the RAGT and the conventional gait training.

**Results:**

The 2 independent factors with the greatest impact on the response to RAGT for the achievement of independent gait were initial cognitive function and affected lower extremity power (*p* < 0.05). However, in the control group, stroke duration from onset to treatment and affected lower extremity power were significant independent factors (*p* < 0.05).

**Conclusion:**

This prespecified analysis suggests that the efficacy of RAGT with a wearable exoskeleton appears to be less dependent on time since onset within the early subacute phase, highlighting the importance of preserved cognitive function.

## LAY ABSTRACT

Stroke survivors often need help learning to walk again. This study compared conventional and an exoskeletal wearable robot-based gait training to see what helps patients walk independently after treatment. We found that having some baseline leg strength was important for both types of therapy. But the other keys to success were quite different. When it comes to conventional gait training, it is really important to start the exercises as soon as possible after the stroke. For patients using an exoskeletal wearable robot, the time since their stroke was less important. Instead, it was their good cognitive function that made them successful. This suggests that using an exoskeletal wearable robot to train their gait could be a great option for stroke survivors to recover, even if it has been a while since their stroke. However, it is important to make sure the patient has the cognitive ability to actively use the technology.

Robot-assisted gait training (RAGT) has emerged as a pivotal therapeutic intervention in neurorehabilitation since its introduction in the late 1990s, originally having been developed to overcome the labour-intensive limitations of manual partial bodyweight-supported treadmill training. By facilitating high-intensity, repetitive, and task-specific practice with consistent kinematic guidance, RAGT allows for a dosage of training that far exceeds what is typically feasible with conventional physical therapy (1, 2). Extensive research supports its efficacy; notably, a recent review established that a treadmill-based RAGT, in combination with physiotherapy, significantly increases the odds of independent walking recovery, particularly in non-ambulatory patients at the subacute stage (3). Furthermore, clinical trials suggest that the “window of opportunity” for RAGT is optimal within the early subacute period, where heightened neuroplastic potential allows for more effective motor relearning compared with the chronic phase (4).

Despite the clinical utility of treadmill-based RAGT, it has been noted that this approach differs from the conditions of actual ground walking (5) and that it can be difficult to align the robot’s movements with the patient’s muscular contraction efforts or the passive characteristics of the musculoskeletal system (6). Consequently, overground gait training using an exoskeleton has recently been proposed as an alternative form of RAGT. This RAGT has been proposed to promote the activation of the nervous system by inducing active participation from the patient who performed active balance control, weight shift, and muscle activation (6).

Recently, we reported the effect of overground gait training using a torque-assisted exoskeleton in patients with subacute stroke on the recovery of ambulatory function (7). Our previous study revealed that there was no significant difference in improving ambulatory function between overground gait training using a torque-assisted exoskeleton and conventional gait training. Nevertheless, given that both groups exhibited enhancements in ambulatory function, the identification of the factors that contribute to the effectiveness of each type of gait rehabilitation could determine the indications for each approach. Therefore, the aim of the current study was to identify factors associated with the achievement of independent gait after the RAGT with an exoskeletal wearable robot in subacute stroke patients. In this analysis, we investigated the characteristics of patients with subacute stroke for the appropriate indications for an exoskeleton in patients with subacute stroke through this prespecified analysis. The identification of the appropriate indication of RAGT with an exoskeleton will aid in the design of individual treatment plans and the accurate stratification of patients for better outcomes after RAGT in patients with subacute stroke.

## METHODS

### Study design

This was a prespecified analysis of an international, multicentre, randomized, controlled trial with blinded outcome evaluation in patients with subacute stroke (8). The study was conducted in 4 hospitals in the Republic of Korea and 2 hospitals in Malaysia. The trial was registered at ClinicalTrials.gov (NCT05157347). The protocol (8) was approved by the Institutional Review Board (IRB) of each hospital (IRB of Severance Hospital, South Korea (IRB no. 1-2021-0031), IRB of National Traffic Injury Rehabilitation Hospital (No. NTRH-21016), IRB of Samsung Medical Center (IRB no. 2021-07-021), IRB of the National Health Insurance Service Ilsan Hospital (No. NHIS- 2021-07-029), and IRB of the Universiti Teknologi MARA (No. REC/04/2021 (MR/26)), and conforms to the Declaration of Helsinki. All participants provided written informed consent before starting the study procedures.

Participants were randomly assigned, in a 1:1 ratio, to the RAGT group and the control group. After randomization, each participant attended a total of 20 sessions, 5 times a week, over 4 weeks. The RAGT group underwent 30 min of conventional gait training and an additional 30 min (excluding robot attachment and detachment time) of gait training utilizing an exoskeleton (ANGEL LEGS M20, Angel Robotics, Co, Ltd) in the physiotherapy room. The control group received conventional gait training for the same duration of 60 minas the RAGT group in the physiotherapy room.

### Participants

**Fig. 1:**
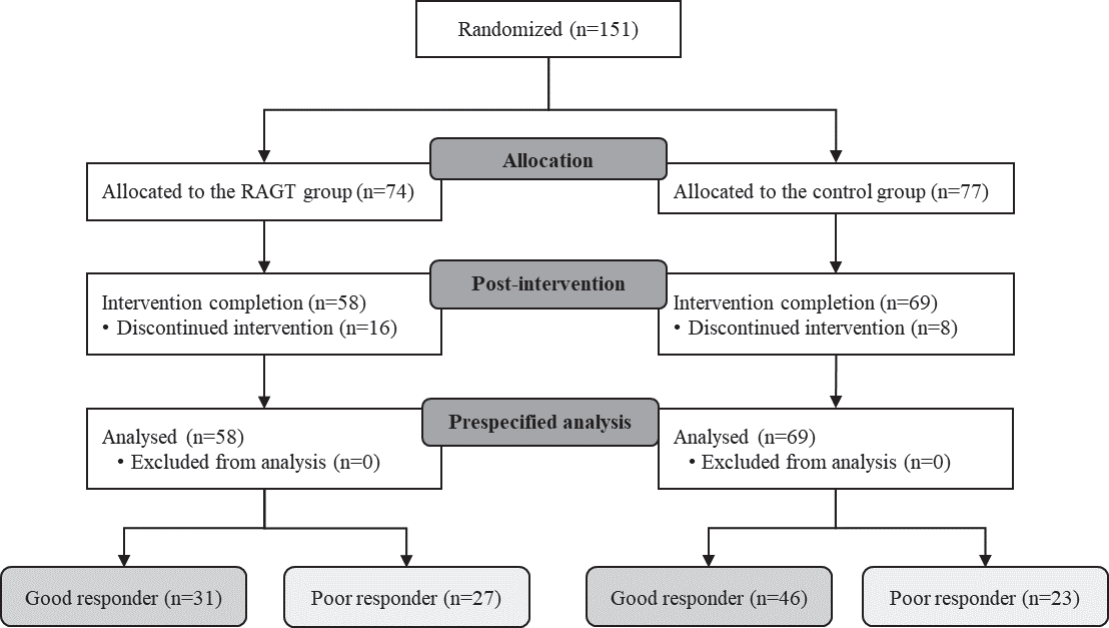
Flow diagram of a prespecified analysis.

A total of 151 patients with subacute stroke were recruited. Of these, 58 participants were randomized in the RAGT group and 69 in the control group were included in the this prespecified analysis (Fig. 1) (7).

The study enrolled adult patients (aged ≥ 19 years) presenting with hemiparesis following either ischaemic or haemorrhagic stroke. Participants were recruited during the early subacute stage, defined as the period from 7 days to less than 3 months post-onset (9). To be eligible, patients must have been capable of independent ambulation without significant disability prior to the stroke (modified Rankin Scale score ≤ 1) (10). At the time of enrolment, participants were required to demonstrate difficulty with independent gait, defined by a Functional Ambulation Categories (FAC) score of ≤ 2, while maintaining adequate trunk control (Trunk Control Test [TCT] score ≥ 50) (11). Additionally, participants had to meet specific physical criteria to ensure compatibility with the wearable robot: height between 140 cm and 190 cm, weight less than 80 kg, and foot length between 230 mm and 290 mm.

Patients were excluded if they presented with significant difficulties in communication, such as severe cognitive impairment (Mini-Mental State Examination score < 10) (12) or severe speech–language impairment. Other neurological exclusion criteria included ataxia resulting from cerebellar pathway lesions, moderate-to-severe spasticity of the affected lower extremity (Modified Ashworth Scale score ≥ 2) (13), or concurrent neurological disorders affecting ambulatory function (e.g., Parkinson’s disease, multiple sclerosis). Furthermore, patients were ineligible if they had severe lower limb musculoskeletal disorders, contractures limiting range of motion, or an apparent leg length discrepancy of 2 cm or more.

### Prespecified analysis

The prespecified analysis evaluated the relationship between potential influencing factors and the achievement of independent gait immediately after the intervention for 4 weeks in each group. Patients were stratified into good and poor responders, defined as whether patients achieved an independent gait. This was defined as the ability to ambulate independently for a distance exceeding 10 m without physical contact, though guidance or monitoring was permitted. This capability was categorized as an FAC score of greater than 3.

The potential influencing factors were selected for this prespecified analysis because they have been found to have some predictive value in previous studies on gait recovery in stroke rehabilitation. For the baseline descriptive characteristics, age, sex, body mass index (BMI), and time since stroke onset were recorded at baseline in each patient (14–17).

At baseline, FAC (18) was used to assess ambulatory function, and the Fugl-Meyer Assessment-Lower Extremity (FMA-LE) (19) and the lower limb score of Motricity Index (MI-LL) (20) were utilized to assess the motor function of the affected lower limb. The assessment of balance function was conducted by TCT and the Berg Balance Score (BBS) (21). In addition, all participants completed self-administered questionnaires with the Geriatric Depression Scale-short form (GDS-SF) (22) and EuroQol-5D (EQ-5D) (23) to assess mood and quality of life at baseline.

### Statistical analysis

SPSS version 29.0 (IBM Corp, Armonk, NY, USA) was used for all statistical analyses. The objective of this prespecified analysis was to establish appropriate clinical indications for RAGT by identifying the specific patient subgroups that respond optimally to RAGT and conventional gait training, respectively. Consequently, both groups were subjected to identical analytical methods, and their respective predictive factors were subsequently compared. Good responder and poor responder groups were compared using an independent *t*-test and χ^2^ tests for normally distributed variables and Mann–Whitney tests for nonparametric data. No significant outliers were found in the analysis of continuous potential influencing factors. To account for potential multicollinearity among the functional variables (TCT, BBS, FMA-LE, and MI-LL), we employed a multivariate binary logistic regression analysis utilizing the backward elimination method to select the most appropriate independent predictors. Univariate binary logistic regression was conducted to identify possible factors for identifying a good responder to each RAGT and control group and any variables with univariate associations with *p*-values < 0.20 were considered to be potentially associated with the intervention and were included in a multivariate model. Multivariate binary logistic regression models utilizing the backward elimination method were then developed (24). A *p*-value < 0.05 was considered to be statistically significant.

## RESULTS

### Comparison between good and poor responders groups

Baseline characteristics were comparable between the RAGT group and the control group, although the RAGT group exhibited a significantly lower FAC *(p* < 0.05). Of the 58 patients in the RAGT group and 69 in the control group, 31 (53%) and 46 (67%) patients who reached the independent gait of FAC (≥ 3) were classified as good responders. In contrast, 27 (46%) and 23 (33%) patients were classified as poor responders in the RAGT group and the control group, respectively. There was no significant difference in the rate of the achievement of independent gait between the 2 groups (*p* = 0.147).

In the RAGT group, FAC, K-MMSE, FMA-LE, and MI-LL at baseline were significantly higher in good responders than in poor responders (*p* < 0.05). In the control group, FAC, BBS and FMA-LE at baseline were significantly higher in good responders than in poor responders (*p* < 0.05). In addition, there was a significantly longer duration in good responders than poor responders in the control group (*p* < 0.05, Table I).

**Table I:** Baseline characteristics of the patients with independent gait and non-independent gait in the robot-assisted gait training group and the control group

	RAGT group (*n* = 58)			Control group (*n* = 69)		
	Independent gait (*n* = 31)	Non-independent gait (*n* = 27)	*p*-value	Independent gait(*n* = 46)	Non-independent gait (*n* = 23)	*p*-value
Demographic characteristics						
Sex (M:F)	22:9	14:13	0.178	25:21	21:13	0.450
Age (years)	60.5 (15.0)	62.2 (13.5)	0.660	57.2 (11.7)	62.8 (15.2)	0.097
Height (cm)	164.7 (8.9)	162.2 (6.8)	0.255	165.6 (8.4)	163.3 (9.2)	0.305
Weight (kg)	64.1 (8.1)	62.9 (9.8)	0.626	63.5 (10.0)	58.8 (8.2)	0.057
Body mass index	23.7 (2.8)	23.9 (3.6)	0.773	23.1 (3.0)	22.1 (2.8)	0.168
Hypertension (yes)	19	17	1.000	25	21	1.000
Diabetes mellitus (yes)	13	5	0.087	17	29	0.276
Heart failure (yes)	0	0	1.000	0	0	1.000
Stroke type (ischaemic:haemorrhage)	20:11	19:8	0.781	28:18	11:12	0.318
Stroke lesion (supratentorial: infratentorial:both)	24:6:1	18:7:2	0.608	41:4:1	18:3:2	0.368
Stroke duration (days)	25.2 (20.3)	34.7 (23.7)	0.103	27.7 (19.9)*	41.4 (23.9)	0.015
Functional characteristics						
FAC	1 [0–2]*	0 [0–2]	0.020	1 [0–2]*	0 [0–2]	0.047
K-MMSE	26.6 (3.5)*	23.0 (6.1)	0.025	23.8 (6.8)	23.5 (4.7)	0.141
TCT	77.5 (15.9)	69.7 (13.2)	0.096	76.7 (16.0)	69.8 (14.1)	0.134
BBS	13.7 (12.2)	8.4 (6.5)	0.122	16.0 (13.8)*	8.9 (9.8)	0.024
FMA-LE	18.3 (7.0)*	13.3 (6.6)	0.003	19.3 (7.6)*	13.8 (8.8)	0.011
MI-LL	49.0 (18.2)*	38.3 (16.6)	0.029	51.3 (19.9)*	37.0 (15.3)	0.003
GDS-SF	7.5 (4.4)	7.3 (4.0)	0.888	6.1 (3.7)	7.3 (4.7)	0.297
EQ-5D	0.4692 (0.2683)	0.4693 (0.2311)	0.950	0.5347 (0.2581)	0.4546 (0.2635)	0.215

Values are presented as number or mean (SD).

RAGT: robot-assisted gait training; FAC: functional ambulatory category; K-MMSE: Korean Mini Mental State Examination; TCT: Trunk Control Test; BBS: Berg Balance Scale; FMA-LE: Fugl-Meyer Assessment-Lower Extremity; MI-LL: lower limb score of Motricity Index; GDS-SF: Geriatric Depression Scale-Short Form: EQ-5D: EuroQal-5D-3L.

* *p* < 0.05, compared with non-independent gait.

### Influencing factors analysis

Table II presents the results of the univariate and multivariate analysis. In the univariate analysis, stroke duration, FAC, K-MMSE, TCT, BBS, FMA-LE, and MI-LL demonstrated potential associations with the achievement of independent gait in the RAGT group (*p* < 0.20). On the other hand, the relatively relating factors in the control group were stroke duration, FAC, TCT, BBS, FMA_LE, and MI-LL (*p* < 0.20). Potential influencing factors with a *p*-value < 0.2 were then used in the multivariate analysis.

In the multivariate analysis, K-MMSE and MI-LL were significantly independent factors to predict good responders in the RAGT group (*p* < 0.05, Nagelkerke’s R_2_ of 0.389). On the other hand, stroke duration and MI-LL were significantly independent factors to predict good responders in the control group (*p* < 0.05, Nagelkerke’s R^2^ of 0.411, see Table II).

**Table II:** Binary logistic regression analysis of the association between baseline clinical features with the patients with independent gait in the robot-assisted gait training group and the control group

Potential influencing factors	RAGT group				Control group			
	Univariate analysis		Multivariate analysis		Univariate analysis		Multivariate analysis	
	Exp(β) (95% CI)	*p*-value	Exp(β) (95% CI)	*p*-value	Exp(β) (95% CI)	*p*-value	Exp(β) (95% CI)	*p*-value
Demographic characteristics								
Sex	–0.820 (0.149–1.300)	0.138	NT		–0.646 (–0.236~1.771)	0.396		
Age	–0.992 (0.986~1.029)	0.654			–0.965 (–0.925~1.007)	0.100	NT	
Body mass index	0.975 (0.826~1.152)	0.768			1.132 (0.948~1.351)	0.170	NT	
Stroke type	0.766 (–0.253~2.314)	0.636			1.697 (0.618~4.659)	0.305		
Stroke duration	0.980 (0.956~1.005)	0.110	NT		0.972 (0.949~0.996)*	0.021	0.968 (0.940~0.997)*	0.031
Functional characteristics								
FAC	3.319 (1.218~9.045)*	0.019	NT		2.090 (1.004~4.351)*	0.049	NT	
K-MMSE	1.168 (1.031~1.325)*	0.015	1.196 (1.044~1.370)	0.010	1.010 (0.931~1.095)	0.813		
TCT	1.038 (1.000~1.078)	0.051	NT		1.031 (0.995~1.068)	0.088	NT	
BBS	1.060 (0.998~1.125)	0.059	NT		1.053 (1.003~1.106)*	0.039	NT	
FMA-LE	1.115 (1.023~1.215)*	0.014	NT		1.089 (1.018~1.165)*	0.014	NT	
MI-LL	1.037 (1.003~1.072)*	0.030	1.045 (1007~1.085)	0.020	1.044 (1.012~1.077)*	0.007	1.050 (1.008~1.094)*	0.018
GDS-SF	1.013 (0.895~1.148)	0.837			0.924 (0.815~1.048)	0.217		
EQ-5D	0.997 (0.124~8.031)	0.998			3.241 (0.476~22.087)	0.230		

RAGT: robot-assisted gait training; FAC: functional ambulatory category; K-MMSE: Korean Mini Mental State Examination; TCT: Trunk Control Test; BBS: Berg Balance Scale; FMA-LE: Fugl-Meyer Assessment-Lower Extremity; MI-LL: lower limb score of Motricity Index; GDS-SF: Geriatric Depression Scale-Short Form: EQ-5D: EuroQal-5D-3L.

* *p* < 0.05.

## DISCUSSION

This prespecified analysis suggest that the overground RAGT with an exoskeletal wearable robot might be expected to be effective in patients with subacute stroke who have relatively good cognitive function despite a relatively longer stroke duration.

In this study, we found previously reported potential factors influencing ambulatory functional recovery after stroke, because interactions between them are likely and might affect recovery. The recovery of gait function in subacute stroke patients is determined by multifactorial interplay involving baseline stroke severity, initial motor and balance deficits, and cognitive and psychosocial status (25). Specifically, previous meta-analyses have identified stroke duration, TCT, and ML-LL as critical factors in this regard (26). The finding that stroke duration and motricity index were found to be independently significant predictors of independent walking in the control group that received conventional gait rehabilitation therapy in this study is considered to be consistent with existing research. The absence of TCT analysis as a significant predictor in this study is hypothesized to be attributable to the restriction of the study’s participants to patients with relatively preserved trunk control function, all of whom had TCT scores of 50 or higher. In contrast to the group that received conventional gait rehabilitation therapy, ML-LL was found to be an independently significant predictor in the RAGT group that received overground gait training using a wearable exoskeletal robot, similar to the control group. However, cognitive function assessed by K-MMSE was identified as another independently significant predictor, and the exclusion of stroke duration is considered a very meaningful result. Cognitive function is crucial for gait recovery because walking is a complex task demanding attention and executive resources to adapt to environmental changes and perform motor learning (27). A previous study (28) reported a significant correlation between technology acceptance and consistent participation in training, and improved performance. As such, robot-assisted gait training necessitates a higher level of technology acceptance among participants in comparison with traditional gait rehabilitation methodologies. These findings may also be considered a limitation of overground gait training using wearable exoskeletons. The provision of suitable feedback in RAGT is regarded as a domain requiring enhancement to facilitate more effective comprehension and active involvement in robot-assisted gait training by participants.

The exclusion of stroke duration as a significant predictor in the RAGT group is considered a highly interesting result. Stroke duration is a critical determinant for the improvement of gait function because the recovery follows a non-linear, time-dependent trajectory driven by biological mechanisms (29). Because conventional gait rehabilitation is based on activity-dependent plasticity driven by repetitive practice, homeostatic neuroplasticity serves as a crucial mechanism to stabilize neuronal excitability and maintain network equilibrium (30). Therefore, to enhance gait function through the mechanism of homeostatic neuroplasticity, intensive gait rehabilitation should be conducted as early as possible during the subacute stroke phase. These results suggest that stroke duration emerged as a significant negative independent predictor in the control group. In contrast, overground gait training using a wearable exoskeletal robot involves a much higher exercise intensity compared with conventional gait rehabilitation. Consequently, it operates primarily through Hebbian neuroplasticity, achieved by performing correct, repetitive, and active tasks (31, 32). As Hebbian neuroplasticity is largely driven by the intensity and repetition of practice, the specific timing of intervention may have a less restrictive influence compared with spontaneous biological recovery. This mechanism may explain why stroke duration did not emerge as a significant predictor in the RAGT group. Timing remains a critical determinant in treadmill-based RAGT. Recent evidence indicates that patients in the subacute phase demonstrate enhanced neuroplastic responses facilitating functional recovery (33), whereas chronic patients can still achieve meaningful gait improvements, though this often necessitates higher intensity or extended intervention periods to induce changes (3). The findings from previous treadmill-based RAGT studies and this study provide highly significant implications for selecting subjects for overground gait training using wearable exoskeletal robots.

### Limitations

A limitation of this study is that it restricted the definition of improved walking function to cases where independent walking was achieved through FAC. When assessing ambulatory patients, it is imperative to consider the normalization of their gait patterns, as opposed to merely evaluating their independent performance. Even in stroke survivors who achieve independent ambulation, persistent gait asymmetry and compensatory kinematic deviations can lead to significantly increased metabolic energy expenditure and secondary musculoskeletal complications, which ultimately limit long-term community participation and increase the risk of falls (34, 35). Consequently, gait pattern considerations assume particular significance in the context of gait rehabilitation during the subacute stroke phase. In addition, given that all subjects were limited to patients with relatively good balance function (TCT ≥ 50), it was not possible to extrapolate the indications for overground gait training using wearable exoskeletal robots to subacute stroke patients with poor balance function. Furthermore, the restriction of the study’s participants to early subacute stroke patients precluded the assessment of the effectiveness of the intervention in stroke patients beyond 3 months post-onset. The finding that stroke duration was not a significant predictor in the RAGT group suggests that overground gait training may offer an extended therapeutic window beyond the early subacute phase. While this study focused on subacute patients, these results support the need for future investigations into the efficacy of this intervention for chronic stroke patients. Consequently, further research is warranted to investigate the underlying mechanisms and potential therapeutic applications of these findings.

### Conclusion

The findings of this study suggest that suitable candidates for the overground RAGT using a wearable exoskeletal robot could be early subacute stroke patients (less than 3 months post-onset) with preserved cognitive function, regardless of stroke duration within this timeframe. It is imperative that further research be conducted to ascertain the practical applicability of these findings.
